# Supplementary material for: Evaluation and screening of technology start-ups based on PCA and GA-BPNN
Source: PLoS One. 2024 Feb 8;19(2):e0289691. doi: 10.1371/journal.pone.0289691 (PMC10852304; doi:10.1371/journal.pone.0289691)
Supplement: Attachment — Submitted filename: Response-7.9.doc [file pone.0289691.s003.doc]

**Response to the Reviewers’ Comments**

On behalf of all the contributing authors, I would like to express our sincere appreciations of your letter and reviewers’ constructive comments concerning our article entitled “Evaluation and Screening of Technology Start-ups Based on PCA and GA-BPNN” (EMID:ac5e6676202ab79f). These comments are all valuable and helpful for improving our article. According to the reviewers’ comments, we have made extensive modifications to our manuscript to make our results convincing. In this revised version, changes to our manuscript were all highlighted within the document by using red-colored text. Point-by-point responses to the two nice reviewers are listed below this letter.

**Reviewer #1:**

1. **How to choose the lag order of the inputs?**

Thank you for the suggestion. The BPNN model consists mainly of an input layer, an implicit layer and an output layer. The number of input and output layers is determined according to the actual problem, and a back-propagation algorithm of errors is used in learning and training, where the data is propagated layer by layer and the weights are continuously corrected so that the mean squared deviation of the output value from the true value meets the expectation. The number of neurons in the input layer of the model is equal to the number of input variables in the data to be processed, and the number of neurons in the output layer is equal to the number of outputs associated with each input. Instead of focusing on the lag order of the inputs, the model is initialized to determine the connections between the input, implicit and output layers in the initial state, the weight values, the threshold values for the implicit layer and the threshold values for the output layer.

2. **Review of the literature can be improved further, may include the study of other applications of BPNN model**

(<https://doi.org/10.1016/j.dsm.2021.10.002>; <https://doi.org/10.1016/j.energy.2022.123990>).

Thank you for the suggestion. We have checked the literature carefully and added the above references into the literature review part in the revised manuscript. (Lines 19-20, page 4)

3. **Add several figures, such as method diagrams, result comparison diagrams, etc.**

Thank you for the suggestion. We have added the convergence curves of the GA-BPNN algorithm and the traditional BPNN (as shown in Figure 4).

4. **The formula is in image format, please change it.**

Thanks for your careful checks. We are sorry for our carelessness. Based on your comments, we have made the corrections.

5. **Why choose GA to optimize BPNN instead of other evolutionary algorithms?**

The genetic algorithm has many advantages over other evolutionary algorithms: the GA is scalable and has a high degree of adaptability in combination with the BPNN model. At the same time, the search is inspired using evaluation functions and the process is simple. Moreover, the use of a probabilistic mechanism for iteration is stochastic and the search starts from a population, which is potentially parallel and allows for the simultaneous comparison of multiple individuals. In view of the shortcomings of BP algorithm, this paper adopts GA to optimize it, uses the advantages of GA for global optimization, to take its optimal solution as the initial weights and thresholds of BPNNM, and obtains the GA-BPNNM.

**Reviewer #2:**

1. **Introduction can be improved. The scientific problem of this research can be described more clearly. Some real-world examples can be added to specify the research problem.**

Thank you very much for your advice. This paper attempts to build an algorithm to reduce the cost of selection of investment targets for venture capitalists, in an attempt to solve the problem of adverse selection of startups that is common among venture capitalists in the investment process. We have amended the corresponding part of the text to make it clearer and more precise. (Lines 23-25, page 2)

2. **The clarity of the formula should be improved.**

Thank you for the suggestion. Modified throughout the paper according to the comment.

3. **Literature review should be improved. Since this study adopt neural network and optimization algorithm. Researches about machine learning models and optimization techniques should be discussed. The following articles should be added.**

[1]<https://www.sciencedirect.com/science/article/abs/pii/S0020025523005091>

[2]<https://www.sciencedirect.com/science/article/abs/pii/S0020025522006260>

[3]<https://www.sciencedirect.com/science/article/pii/S2666764921000485>

[4]<https://www.sciencedirect.com/science/article/pii/S2666764922000364>

[5]<https://www.sciencedirect.com/science/article/abs/pii/S0957417422013057>

[6]<https://www.sciencedirect.com/science/article/abs/pii/S0957417421000129>

We sincerely appreciate the valuable comments. We have checked the literature carefully and added the above references into the literature review part in the revised manuscript. (Lines 10-30, page 4)

4. **The training result of BPNNM and GA-BPNNM can be presented and compared. For example, the Convergence curve when training the model.**

Thank you for the suggestion. We have added the convergence curves of the GA-BPNN algorithm and the traditional BPNN (as shown in Figure 4, page 23).

5. **The effectiveness of PCA should be evaluated by comparing the model without using PCA.**

Thank you very much for your advice. We are sorry for the misunderstanding as we did not express it clearly. The use of PCA is necessary because the complexity and diversity of the influencing factors for start-ups requires the data related to all influencing factors to be downscaled. The use of PCA is necessary to filter and identify a total of 22 input variables, a necessary part of the determination of the number of input layers based on reality, to make the model more scientific.

6. **English quality should be improved.**

Thanks for your suggestion. We have invited a native English speaker from the USA to help polish our article. And we hope the revised manuscript could be acceptable for you.

According to the reviewer’s comments, we have revised the manuscript extensively. If there are any other modifications we could make, we would like very much to modify them and we really appreciate your help. We hope that our manuscript could be considered for publication in your journal. Thank you very much for your help.
