# Supplementary material for: Evaluation and screening of technology start-ups based on PCA and GA-BPNN
Source: PLoS One. 2024 Feb 8;19(2):e0289691. doi: 10.1371/journal.pone.0289691 (PMC10852304; doi:10.1371/journal.pone.0289691)
Supplement: Attachment — Submitted filename: Response to Reviewers-30NOV.doc [file pone.0289691.s004.doc]

**Answers for the Reviewers’ Comments**

On behalf of all the contributing authors, I would like to express our sincere appreciations of your letter and reviewers’ constructive comments concerning our article entitled “Evaluation and Screening of Technology Start-ups Based on PCA and GA-BPNN” (EMID: ac5e6676202ab79f). These comments are all valuable and helpful for improving our article. According to the reviewers’ comments, we have made extensive modifications to our manuscript to make our results convincing. In this revised version, changes to our manuscript were all highlighted within the document by using yellow-colored text. Point-by-point responses to the nice reviewers are listed below this letter.

**Reviewer #1: The author has revised my comments. Future revisions need to have the changes highlighted for easier review.**

We sincerely appreciate the valuable comments. We’ve highlighted the changes in yellow to make it easier to review.

**Reviewer #3:**

1. **Please refer to “Table 1. (Technology) start-ups/ project assessment and evaluation index system”. Since based on a review of the literature the indicators are already finalized, “hence, Etc.” may be removed under the first level indicators.**

Thank you very much for your advice. We have amended the corresponding part of the first level indicators to make it clearer and more precise. (Table 1, page 6)

2. **Please refer to “Using the Delphi method, through 6 experts and 9 investment practitioners and 6 rounds of anonymous discussion and communication, the final evaluation system and index score for TSs are determined, including 4 first-level indicators, 15 second-level indicators and 33 third-level indicators.” The role of Delphi is crucial in the decision-making, how the selection of Delphi was carried out, their background and process of 6 rounds is crucial as it affects the outcome. Authors may provide more information on it.**

Thank you for your valuable comments on the construction of the indicator system. We fully agree with the reviewer’s suggestions for modifications and acknowledge the lack of description of the implementation process of the Delphi method. We have added detailed implementation processes of the Delphi method to the manuscript, including the background characteristics of experts in the Delphi method and the specific implementation process. The newly added sections in the manuscript are as follows:

We utilized the Delphi method to further determine the indicators and their weights. Specifically, we selected six professors from universities and nine managers from related companies. All respondents were over 30 years old. Five professors from universities were all doctoral supervisors, and their research directions were all business management. The nine managers had more than five years of experience in technology investment companies or technology platform companies. Through online meetings, we solicited opinions from the aforementioned experts, distributed survey outlines and background resources in advance, and answered some experts’ queries. We summarized the experts’ first-round discussion opinions on the indicator system and the results to the experts for the second revision. After the fourth round of anonymous discussion, the indicator system was finally determined, and the experts’ opinions were unified. In the subsequent fifth and sixth rounds of discussion, we asked the experts to provide importance evaluations for the three-level indicators of the determined indicator system, thus further determining the corresponding scores for each indicator. The final evaluation system and index scores for TSs include 4 first-level indicators, 15 second-level indicators, and 33 third-level indicators. As shown in Table 2.

We thank the reviewer for his or her feedback and are pleased to have had the opportunity to address the issues raised. We hope that our additional explanations and clarifications have addressed the concerns raised in a satisfactory manner.

3. **Please refer to Table 2. Evaluation index system of TSs, various points are included in Table 2, the basis of allocation of these points and their use is unclear. The points may be written in a column adjacent to the “Code” column.**

Thank you for your interest in the indicator system. We need to explain the source of the scores in Table 2. The scores for each indicator in Table 2 were obtained through the Delphi method. We conducted six rounds of Delphi discussions, with the first four rounds determining the indicator system, including the three-level indicators. In the fifth and sixth rounds, we asked experts to provide importance evaluations for each indicator in the indicator system, that is, to determine the corresponding scores for the indicators. Through two rounds of iterative discussions, we finally determined the corresponding scores for the three-level indicators. We have also added the implementation process of the Delphi method and the source of the indicator scores to the manuscript. The newly added sections in the manuscript are shown below：In the subsequent fifth and sixth rounds of discussion, we asked the experts to provide importance evaluations for the three-level indicators of the determined indicator system, thus further determining the corresponding scores for each indicator.

4. **Please refer to “2.3 Data source”: what was the basis of “The selection criteria are as follows: “The data source was gathered through the questionnaire as mentioned in”. One is through questionnaires that were filled out by these LTSs, and another is from information publicly disclosed by them.” may be uploaded as a supplementary file along with collected data file.**

Thank you for your valuable comments. We apologize for any misunderstandings. After a collective discussion among the authors and a re-examination of the original data, we discovered that there may have been some misunderstanding regarding the term **“**questionnaire**”** in the original manuscript. It should be clarified that we used the Delphi method to determine the indicators and weights, following which we collected the data through public disclosure and submitted the relevant data sets and codes as supplementary materials. In reality, the Delphi method is vastly different from the conventional questionnaire survey. In conclusion, this study’s data sources do not include data from questionnaire surveys, and we have revised the content of the manuscript accordingly. I deeply apologize for any misunderstandings caused by the previous incorrect expression.

5. **The author may define qualified enterprises and unqualified enterprises also clarify the basis of classification, “Finally, data of 200 sample start-ups are obtained, and among these samples, 100 start-ups are qualified enterprises, and 100 unqualified.”**

Thank you very much for your advice. We are sorry for the misunderstanding as we did not express it clearly. We rechecked the data sources section of the manuscript and found that more discussion on qualified firms was missing in this section. Therefore, we add the classification criteria of qualified enterprises and unqualified enterprises in Section 2.3.

6. **The questionnaire pilot testing, questionnaire validity and response accuracy are important in an empirical investigation, which is missing.**

We thank you for your comments. Our study did not obtain data through questionnaire survey. Our goal was to conduct expert discussions using the Delphi method to determine the indicator weights. This is fundamentally different from the traditional approach of using questionnaire surveys to gather enterprise data for analysis, and this method of determining the weights of indicators is similar to the entropy weight method.

The Delphi method is a structured consulting technique that reflects the collective opinion of experts, and it does not require additional reliability or validity testing. We recognize that it may have been unclear in the manuscript how the Delphi method was implemented, which may have led to this misconception. Therefore, we have included more detailed information on expert selection, the decision-making process of the Delphi method, and so on, to supplement the manuscript.

7. **Please refer to make the results obtained more scientific and accurate for this evaluation system, the PCA method is used for the final sample data to extract the principal components and eliminate the multicollinearity between indicators. The SPSS outcomes for factor loading in the manuscript may be provided.**

We greatly appreciate your comments. Upon examination of the manuscript, we noticed that the presentation of the results obtained through the principal component analysis method was not comprehensive enough. Therefore, in section 3.2, we have included more detailed results of the principal component analysis, including formulas and factor load calculations.

8. **There are some typos in the manuscript, for instance refer to the abstract: under purpose, the text is in mixed font sizes may be corrected.**

Thanks for your careful checks. We are sorry for our carelessness. Based on your comments, we have checked the literature carefully and made the corrections.
