# Supplementary material for: Evaluation and screening of technology start-ups based on PCA and GA-BPNN
Source: PLoS One. 2024 Feb 8;19(2):e0289691. doi: 10.1371/journal.pone.0289691 (PMC10852304; doi:10.1371/journal.pone.0289691)
Supplement: Attachment — Submitted filename: Response to Reviewers-11DEC.doc [file pone.0289691.s005.doc]

**Answers for the Reviewers’ Comments**

On behalf of all the contributing authors, I would like to express our sincere appreciations of your letter and reviewers’ constructive comments concerning our article entitled “Evaluation and Screening of Technology Start-ups Based on PCA and GA-BPNN” (EMID: ac5e6676202ab79f). These comments are all valuable and helpful for improving our article. According to the reviewers’ comments, we have made extensive modifications to our manuscript to make our results convincing. In this revised version, changes to our manuscript were all highlighted within the document by using yellow-colored text. Point-by-point responses to the nice reviewer are listed below this letter.

**Reviewer #3: (a) Furthermore, except remark number 6, the most of the comments have been satisfactorily resolved. The explanation appears to be a satisfactory one; nevertheless, a Delphi process content validity index could provide value to the manuscript.**

Thank you for your valuable comments. Due to space constraints, we had not initially included an indicator of content validity for the Delphi process in the manuscript. We thank the reviewer for bringing this to our attention, as we recognize that adding this section would enhance the rigor and scientific soundness of the article while adhering to the consistently high standards expected by the PLOS ONE journals. We have accordingly included the corresponding validity test content. The newly added sections in the manuscript are as follows: Prior to initiating the formal research of this paper, specifically prior to distributing the survey questionnaire, eight experts were requested to evaluate the validity of the questionnaire's structure, content, and overall design. Based on the evaluation results from these eight experts (see Tables 2.2), they provided positive feedback on the questionnaire structure, content, and overall design. Therefore, the survey questionnaire designed in this paper has a high level of validity.

**Table 2.2 Result of experts' evaluation (N=8)**

|  | | Fairly reasonable | Basically sound | Ordinary | Somewhat unreasonable | Completely unreasonable |
| --- | --- | --- | --- | --- | --- | --- |
| Structure | Number of choices | 3 | 5 | 0 | 0 | 0 |
|  | Percentage | 38% | 63% | 0% | 0% | 0% |
| Content | Number of choices | 2 | 6 | 0 | 0 | 0 |
|  | Percentage | 25% | 75% | 0% | 0% | 0% |
| Overall  design | Number of choices | 2 | 6 | 0 | 0 | 0 |
|  | Percentage | 25% | 75% | 0% | 0% | 0% |

**(b) Data provided in the Table 5. Principal component factor load matrix looks incomplete and Table 6. Sample data of TSs look incomplete.**

Thank you very much for your suggestion. We have added the corresponding parts of the principal component coefficient loading matrix to make it clearer and more precise. Limited to space, we have provided table 6 in the annex of the manuscript.

**(c) A complete data file may be uploaded.**

Thank you very much for your advice. We neglected the integrity of the data in the past, which led to a loss of rigor in the manuscript. We have once again verified all the data and additional attachments, and have included complete data as per the commenter's recommendations.
